# Comprehensive Profiling of Surface Gangliosides Extracted from Various Cell Lines by LC-MS/MS

**DOI:** 10.3390/cells8111323

**Published:** 2019-10-26

**Authors:** Jua Lee, Heeyoun Hwang, Sumin Kim, Jaeyun Hwang, Jaekyung Yoon, Dongtan Yin, Sun Il Choi, Yun-Hee Kim, Yong-Sam Kim, Hyun Joo An

**Affiliations:** 1 Graduate School of Analytical Science & Technology, Chungnam National University, Daejeon 34134, Korea; jualeeg@gmail.com (J.L.); ksm6781431@gmail.com (S.K.); hjyuni89@gmail.com (J.H.); yunjaekeung@gmail.com (J.Y.); yindongtan@gmail.com (D.Y.); 2 Asia-Pacific Glycomics Reference Site, Daejeon 34134, Korea; 3 Research Center of Bioconvergence Analysis, Korea Basic Science Institute, Cheongju-si 28119, Korea; heeyounh@kbsi.re.kr; 4 Department of Cancer Biomedical Science, National Cancer Center Graduate School of Cancer Science and Policy, Goyang 10408, Korea; sunvely.choi@gmail.com (S.I.C.); sensia37@ncc.re.kr (Y.-H.K.); 5 Genome Editing Research Center, Korea Research Institute of Bioscience and Biotechnology (KRIBB), Yuseong-gu, Daejeon 34141, Korea; omsys1@kribb.re.kr; 6 Department of Biomolecular Science, KRIBB School of Bioscience, Korea University of Science and Technology (UST), Yuseong-gu, Daejeon 34113, Korea

**Keywords:** glycosylation, ganglioside, cancer cell, cell surface, LC-MS/MS

## Abstract

Gangliosides act as a surface marker at the outer cellular membrane and play key roles in cancer cell invasion and metastasis. Despite the biological importance of gangliosides, they have been still poorly characterized due to the lack of effective analytical tools. Herein, we performed molecular profiling and structural elucidation of intact gangliosides in various cell lines including CFPAC1, A549, NCI-H358, MCF7, and Caski. We identified and quantified a total of 76 gangliosides on cell membrane using C18 LC-MS/MS. Gangliosides found in each cell line exhibited high complexity and diversity both qualitatively and quantitatively. The most abundant species was GM3(d34:1) in CFPAC1, NCI-H358, and MCF7, while GM2(d34:1) and GM1(d34:1) were major components in A549 and Caski, respectively. Notably, glycan moieties showed more diversity between cancer cell lines than ceramide moieties. In addition, noncancerous pancreatic cell line (hTERT/HPNE) could be distinguished by gangliosides containing different levels of sialic acid compared with cancerous pancreatic cell line (CFPAC1). These results clearly demonstrated the feasibility of our analytical platform to comprehensive profile of cell surface gangliosides for identifying cell types and subgrouping cancer cell types.

## 1. Introduction

Cell membrane consists of a lipid bilayer including cholesterols, phospholipids, sphingolipids, and integral and peripheral proteins, where a substantial number of sphingolipids and proteins have been glycosylated [1]. Cell surface glycosylation mainly consists of large biomolecules, glycoproteins, and glycosphingolipids, respectively. Glycosylation, which is highly sensitive to the external environment, has been regarded as a window reflecting physiological state of cell because it plays an important role in multiple cellular activities such as cell differentiation and cell growth [2]. In addition, glyco-conjugates on cell membranes act as communicators, sending and receiving signals from neighboring cells and the environment [3]. Thus, many researchers already have paid attention to glyco-conjugate not only as valuable markers (targets) for identifying the cell as part of the same organism or as foreign but also as hallmarks of cancer [4,5,6]. However, the study of glycosylation to date has focused on glycoproteins rather than glycosphingolipids due to the lack of adequate analytical tools to determine extreme chemical property, amphipathic nature, and the structural complexity of glycosphingolipids [7]. The glycan moiety, which is generated by non-template driven enzymatic reactions has tremendous heterogeneity in not only monosaccharide composition, but also in sequence, branching, and linkage. In addition, the ceramide moiety can possess several hydroxyl groups (2–4) and diverse levels of unsaturation and length of fatty acyl chain.

Ganglioside is a type of glycosphingolipids, which is composed of a ceramide backbone and a glycan head with one or more sialic acids. The ceramide backbone moiety is anchored in the plasma membrane of cells, whereas the glycan head moiety is exposed on the extracellular surface. The gangliosides have been reported to play a key role in cancer cell invasion and metastasis in several types of cancer such as lung cancer [8,9], melanoma [10,11], neuroblastoma [12,13], glioblastoma [14,15], and breast cancer [16,17]. In particular, gangliosides with specific glycan heads were known to be closely related with several types of cancer and disease. For example, GD3 (di-sialo glycan head) and GM2 (mono-sialo glycan head) promote tumor-associated angiogenesis and regulate cell adhesion/motility for the metastasis in melanoma and neuroblastoma [18]. On the other hand, GM1 regulates metastatic potential of lung cancer cell by modulating location and secretion of matrix metalloproteinase-9 and eventually regulates the tumor invasion activity [19]. In addition, GM3 and GT1b are the major common ganglioside in brain metastatic tumor tissues [20]. Furthermore, gangliosides have received substantial attention as an immunotherapeutic target for cancer diagnosis and treatment as well as a cancer biomarker due to their potential relevance in cancer diagnosis and therapeutics. For example, Ravotumomab which is NeuGc-GM3 based vaccine has been tested in clinical trials of phase III for lung, breast, melanoma, and neuroectodermal pediatric tumors [21]. Also, clinical trials of anti-GD2 antibody drugs such as Hu3F8, Dinutuximab, 14G2a, Ch14.18 and Hu 14.18 are ongoing for neuroblastoma [22,23,24].

Nonetheless, of the biological importance of gangliosides, their characterization has been analytically challenging. Conventional approaches to characterize gangliosides which employ thin-layer chromatography (TLC) and immunoassay with antibodies and lectins have several limitations [25]. In addition to low specificity and sensitivity, they provide insufficient information on ganglioside structure since commercially available ganglioside standards and proper antibodies are limited [26]. Furthermore, they could detect and quantify only a few glycan epitopes for which there are known lectins, and nature of the ceramide moiety cannot be revealed [27]. Therefore, to address the complexity of cell surface gangliosides, development of novel and rapid analytical platform is required. Highly sensitive LC/MS technology has recently emerged as an effective tool for characterizing large numbers of intact gangliosides [28,29,30]. However, full characterization of structural complexity of gangliosides is still remained to be addressed.

Herein, the present study combines cell membrane extraction method with nano-LC/MS analysis for comprehensive and comparative identification and quantitation of cell surface gangliosides from various cancer cell lines, CFPAC1, A549, NCI-H358, MCF7, and Caski. We identified over 70 gangliosides from cell lines in total with great confidence by considering accurate mass, tandem MS, and retention time. Using our approach, we determined compositional and structural diversity of surface gangliosides between different cancer cells. Interestingly, lung cancer subtypes, A549 and NCI-H358 were completely distinguished by ganglioside profile. Furthermore, gangliosides were regrouped into glycan and ceramide moieties respectively, and validated to determine the major effect on ganglioside differences between cell lines. Low correlation between cell lines based on glycan moiety compared with ceramide moiety clearly indicates that glycan moiety exhibits higher heterogeneity depending on cell types than ceramide moiety in ganglioside. We have no doubt that our result on comprehensive profiling of surface ganglioside in various cell types suggest that given analytical platform would provide valuable implications on discovery of cancer cell type-specific molecular targets for cancer biomarker and therapeutics.

## 2. Materials and Methods

### 2.1. Cell Culture

Cancer cell lines of A549 (lung cancer), NCI-H358 (lung cancer), MCF7 (breast cancer), Caski (cervical cancer), and CFPAC1 (pancreatic cancer) and a normal pancreatic cell line, hTERT/HPNE were obtained from American Type Culture Collection (ATCC, Rockville, MD, USA). Cell cultures were performed using the ATCC-recommended growth media, subcultivation ratios, and medium renewal interval from ATCC animal cell culture guide which was downloaded from ATCC Web site [31]. During cell harvest, cells were scraped rather than enzymatically lifted. For each cancer cell sample, approximately 20 million cells were counted and collected in duplicate. Additionally, the same numbers of hTERT/HPNE cells were prepared for comparison between normal and pancreatic cancer cells.

### 2.2. Cell Membrane Extraction

Ultracentrifugation was performed for membrane extraction from the cells as followed. The cells were interrupted in a homogenization buffer consisting of 0.25 M sucrose, 20 mM HEPES-KOH (pH 7.4), and 1:100 protease inhibitor mixture (Calbiochem/EMD Chemicals in Merk, Darmstadt, Germany). Then the samples were sonicated on ice. The nuclear fraction of the lysates was pelleted by centrifugation at 1000 g for 10 min, and the supernatant was collected. Following ultracentrifugation at 60,000 rpm for 45 min at 4 °C, the membrane fraction was pelleted. The membrane pellet was washed and re-suspended in 0.2 M Na_2_CO_3_ (pH 11), and finally pelleted once more. The supernatant was removed, and the membrane fractions were frozen at −80 °C.

### 2.3. Purification and Enrichment of Gangliosides from Cell Membrane

For enrichment of ganglioside from cell membranes, the pellets were re-solubilized in 1mL of H_2_O/CHCl_3_/MeOH (3:4:8; v/v/v). The mixture was incubated in a bath sonicator for 30 min at 10 °C and centrifuged at 8800 g for 2 min at 4 °C. After centrifugation, the supernatants were transferred into a new vial. The pellet was re-extracted in the same volume of H_2_O/CHCl_3_/MeOH (3:4:8; v/v/v), CHCl_3_/MeOH (1:1; v/v), and CHCl_3_/MeOH (2:1; v/v). Each supernatant was pooled with that from first extraction, dried by a SpeedVac, and re-suspended in 1 mL of CHCl_3_/MeOH (2:1; v/v). For Folch partitioning [32] to isolate lipid-containing compounds, 200 µL of 0.1 M KCl were added, and the supernatants were centrifuged at 8800 g for 2 min at 4 °C. Then the upper phase was collected, and the lower phase was re-extracted twice with 300 µL of MeOH/0.1 M KCl (1:1; v/v). The pooled sample of all upper phases washed with 500 µL of CHCl_3_/MeOH (2:1; v/v). C18 solid-phase extraction was performed for enrichment of ganglioside. C18-Sep-Pac cartridge (200 mg, 3.0 mL, Waters) was washed with 5 mL of MeOH, 3 mL of CHCl_3_/MeOH (1:1; v/v) and 2 mL of H_2_O. The upper phase solution was loaded onto the C18 cartridges and washed with H_2_O for desalting. Gangliosides were eluted with sequential addition of 5 mL of CHCl_3_/MeOH (1:1; v/v) and 2 mL of H_2_O/CHCl_3_/MeOH (3:4:8; v/v/v). The elutes were dried in a SpeedVac prior to MS analysis.

### 2.4. Negative Nano-LC Chip Q-TOF MS Analysis of Gangliosides

Extracted gangliosides were analyzed by Agilent 6540 nano-LC chip Q-TOF MS system. (Agilent Technologies, Santa Clara, CA, USA). The nano-LC chip was composed of a 40 nL enrichment column, a 43 × 0.075 mm id analytical column packed with ZORBAX C18, and a nano electrospray tip. Separation was carried out by a binary gradient A: H_2_O and B: 15% IPA in MeOH (v/v), both including 20 mM ammonium acetate and 0.1% acetic acid. The capillary pump was operated at 3 μL/min with 70% solvent B for the loading of the sample onto the enrichment column. A gradient-based chromatographic separation was performed on the analytical column, driven by the nano-pump running at 0.3 µL/min. The 60-min gradient was programmed as follows: 1–3 min, 70–80% B; 3–40 min, B increased to 100%, then continued 100% B to 45 min, finally 70% B for 15 min to equilibrate the chip column before next sample injection. The Q-TOF MS was operated in the negative ion detection mode for MS scans. In addition, MS/MS analysis was performed in both negative and positive ion modes. The drying gas temperature and gas flow were 325 °C and 4 L/min, respectively. Recorded mass ranges were *m/z* 500–2500 for MS only and *m/z* 100–3000 for MS/MS. Acquisition rates were 2s/spectra for MS and 0.63 s/spectra for MS/MS. The slope and offset values of the energy-*m/z* ramp could be altered as needed to produce more or less fragmentation. LC/MS data analysis was performed with the Molecular Feature Extractor algorithm included in the MassHunter Qualitative Analysis software (Version B.6.00, Agilent Technologies).

### 2.5. Ganglioside Identification 

Initially, the monoisotopic mass of each compound was calculated with isotopic distribution, charge state information, and retention time using an in-house program to assign ganglioside composition by accurate mass. All ion signals associated with each compound (e.g., the doubly protonated ion, the triply protonated ion, and all associated isotopologues) were summed together to determine compound abundance. Deconvoluted experimental masses were compared against theoretical ganglioside masses using a mass error tolerance of 20 ppm. Gangliosides were finally identified through their *m/z*, retention times (RT) and MS/MS fragmentation pattern. Multivariate analyses of differentially expressed gangliosides were performed with MetaboAnalyst 4.0 by Partial least squares-discriminant analysis (PLS-DA) and unsupervised hierarchical clustering heatmaps as following: After the missing value imputation with half of the minimum value in the data, each quantified intensity of the gangliosides was scaled by log transformed and mean centering, and normalized by quantile normalization method. Differences between the various cell lines were determined by one-way analysis of variance (ANOVA), followed by Fisher’s post hoc test. Then, gangliosides with a *p*-value < 0.05 were used for the hierarchical clustering with Euclidean distance and Ward clustering algorithm.

## 3. Results 

### 3.1. Profiling of Gangliosides by Negative Ion Detection Mode LC/MS 

**Figure 1:**
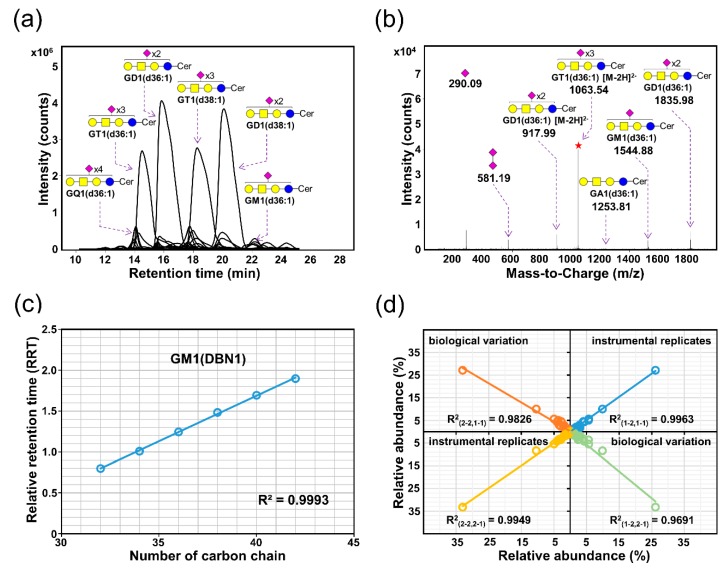
Strategy for identification of cancer cell surface gangliosides. Putative structures of glycan head group were described and ceramide tail was indicated by ‘Cer’. (**a**) Overlaid extracted compound chromatograms (ECCs) of porcine brain ganglioside standard via negative ion mode nano-LC-MS. Peaks of selected gangliosides were annotated with schematic representations of their glycan head group structures and the ceramide species. Monosaccharide legend: Blue circle—Glucose, Yellow circle—Galactose, Yellow square—N-acetylgalactosamine, Purple diamond—N-acetylneuraminic acid. (**b**) Representative tandem MS spectra of ganglioside [GT1(d36:1)] identified from porcine brain with the fragmentation pattern annotated for relevant peaks. Putative structures of the compound are also depicted with designations for product ion peaks. Precursor ion was marked with red star. (**c**) Representative logarithmic relationship existed in GM1 with 1 double bond in their ceramide proportion between ceramide carbon numbers and their retention times on the C18 column. (**d**) Correlations between the ganglioside profiles of NCI-H358 biological replicates and instrumental replicates.

**Figure 2:**
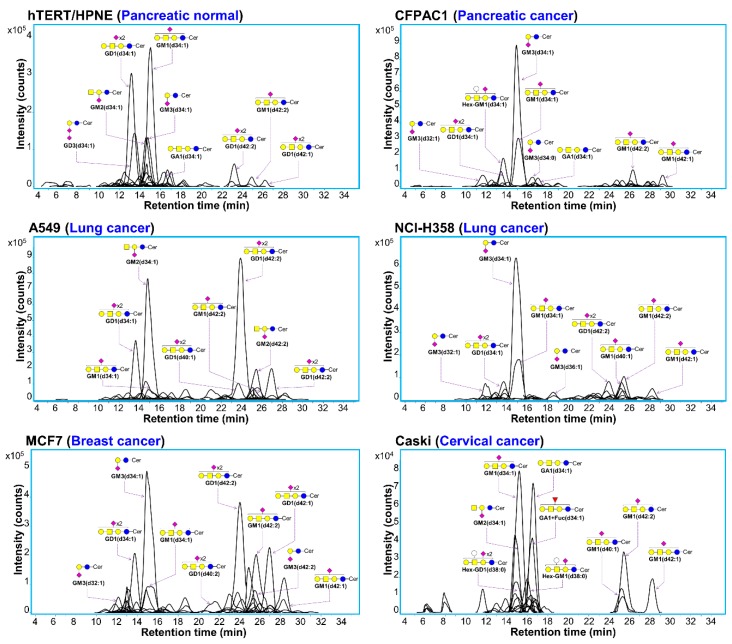
Representative extracted compound chromatograms (ECCs) of cell surface gangliosides detected in different six cell lines. Peaks of major gangliosides were annotated with schematic representations of their glycan head group structures and the ceramide species. Monosaccharide legend: Blue circle—Glucose, Yellow circle—Galactose, Yellow square—N-acetylgalactosamine, Purple diamond—N-acetylneuraminic acid, Red triangle—Fucose. The ceramide tail was indicated by ‘Cer’. The *y*-axis was adjusted to the most abundant ganglioside compound.

Due to the amphipathic property and the structural complexity of gangliosides, well balanced proportions of organic solvents were required for efficient resolving, chromatographic separation, and ionization for mass spectrometry. Prior to the analysis of cancer cell line samples, well-characterized porcine brain gangliosides were analyzed using C18 nano-LC/MS in negative ion detection mode in order to validate our analytical platform. The mixture of 15% isopropanol and methanol (v/v) with gradient, as the organic mobile phase, provided the best chromatographic performance. Figure 1a shows the extracted compound chromatograms (ECCs) of porcine brain gangliosides. The separation of ganglioside was effectively performed on the C18 stationary phase, where over eighty gangliosides were identified from the porcine brain. GM1, GD1 and GT1 with d36:1 and d38:1 were identified in abundance, which is consistent with previous studies [33]. CID tandem mass analysis was further performed to confirm the porcine brain gangliosides. In detail, in addition to intense fragments of acidic mono- and oligo- saccharide including NeuAc (*m/z* 290. 09) and 2NeuAc (*m/z* 581.18), sequential loss of monosaccharide residue from precursor ions were observed in CID tandem MS spectra as shown in Figure 1b, representative example of GT1 (d36:1). Then our LC-MS/MS based analytical platform was applied to investigate gangliosides from plasma membranes of various cancer cell lines such as A549, NCI-H358, MCF7, Caski, and CFPAC1 as shown in Figure S1. Representative ganglioside profiling obtained from MCF7 was shown in Figure S2a,b. We obtained comprehensive information on different surface ganglioside compounds from the cancer cell. The separation of gangliosides on C18 column mainly depends on the ceramide chain length rather than the structure of glycan moiety. Concurrent observation of both high- and low-abundant gangliosides was obtained, covering a dynamic range surpassing 6 orders of magnitude. We could obtain similar observation in chromatographic separation from other cell lines (Figure 2). In common, GA1, GM1, GM2, GM3, GD1, and GD3 were observed as major glycan head in all cancer cells although the most abundant species was quite different between cell lines. Ceramides composed of a sphingoid base and a fatty acid were found to have mostly 32 to 42 total carbon atoms with 1 to 2 hydrocarbon unsaturation. Detailed comparative examination of gangliosides between cell lines will be discussed in Section 3.3 below. 

### 3.2. Identification of Cell Surface Gangliosides 

Cancer cell gangliosides were initially assigned based on the exact masses of possible combinations of glycan and ceramide compositions. Furthermore, we confirmed the assignment by identifying gangliosides using both CID MS/MS and their corresponding retention times on C18 column. To figure out the structure of gangliosides, all fragment ions including glycan such as hexose (glucose and galactose), fucose, N-acetylhexosamine (N-acetylglucosamine and N-acetylgalactosamine), and N-acetylneuraminic acid, ceramide including fragmented sphingosine were considered. Figure S2c,d showed CID tandem MS spectra of GM1(d34:1) obtained by negative and positive ion detection mode from the MCF7 cell line, respectively. As shown in Figure S2c, we could observe the connectivity of monosaccharides within a glycan moiety from negative ion detection mode tandem MS spectra In particular, the presence of acidic glycosphingolipid could be determined by intense diagnostic ion at *m/z* 290.087 [M − H]^−^ corresponding to sialic acid (NeuAc). On the other hand, we could obtain more abundant fragment ions via positive ion detection mode tandem MS/MS. As shown in Figure S2d, in addition to information on glycan connectivity, fragments of ceramide moieties (*m/z* 520.50 corresponding to [d34:1]) including sphingosine base (*m/z* 264.26 corresponding to [d18:1]) were apparently obtained from positive ion detection mode. 

Furthermore, identified gangliosides by LC-MS/MS were reconfirmed using retention time prediction, as described in the literature [28,34]. For example, Figure 1c showed very high relationship (*R^2^* = 0.9993) among GM1 compounds with one double bond in NCI-H358. A relative retention time was calculated by dividing RT of each ganglioside species by RT of GM3 (d34:1), which was selected as reference species due to it’s the highest relative abundance in NCI-H358. Mathematical models of relative retention time (RRT) versus the number of carbons in the ceramide for different glycan headgroups established from each cell line were shown in Figure S3. Particularly, minor gangliosides could be identified with greater confidence by the prediction of retention time by considering separation trends affected by the ceramide chain length and the degree of unsaturation of the alkyl moiety. Good linearity was observed between the number of ceramide carbon and the corresponding retention times for each glycan compounds with identical double bonds in all cell lines (0.998 ≤ *R*^2^ ≤ 1.000). Furthermore, duplicate technical experiments with biological duplicates were performed to examine the reproducibility of the method on a run-to-run basis. Correlation between the relative intensity of ganglioside peaks presented high similarity (*R*^2^ = 0.901–0.996) (Figure 1d). Finally, we generated the complete list of the 76 obvious surface gangliosides with biological and experimental replicates containing composition, mass value, and normalized abundances (Table S1). 

### 3.3. Differences in Ganglioside Profiles between Cell Lines 

Qualitative and quantitative diversity of gangliosides found in each cell line such as hTERT/HPNE, CFPAC1, A549, NCI-H358, MCF7, and Caski could be observed solely in chromatograms (Figure 2). Major abundant gangliosides consisting of early-eluting (before 20 min) and late-eluting (after 20 min) species were quite different in each cell lines. Among early eluting species, GM3 (d34:1) were mainly found in CFPAC1, NCI-H358, and MCF7, while, GM2 (d34:1) and GM1 (d34:1) were mainly presented in A549 and Caski, respectively. Note that, subtypes of lung cancer A549 and NCI-H358 even exhibited significantly different elution chromatographic pattern of gangliosides. Specifically, main early-eluting species were GM2(d34:1) and GD1(d34:1) in A549, while GM3(d34:1) and GM1(d34:1) in NCI-H358. Main late-eluting species of A549 was GD1(d42:2), while only minor species were observed in NCI-H358 at corresponding retention time. In addition, normal pancreatic cell line (hTERT/HPNE) and pancreatic cancer cell line (CFPAC1) showed dramatic diversity in early-eluting species. Major early-eluting molecules were GM1(d34:1) and GD1(d34:1) in hTERT/HPNE, while GM3(d34:1) and GM1(d34:1) in CFPAC1. Thirty-four and forty-two carbon numbers in ceramides were prominently observed (account for approximately 94%, 84%, 80%, 82%, and 83% in CFPAC1, A549, NCI-H358, MCF7, and Caski, respectively), although there was still diversity in minor ceramide portion species. 

**Figure 3:**
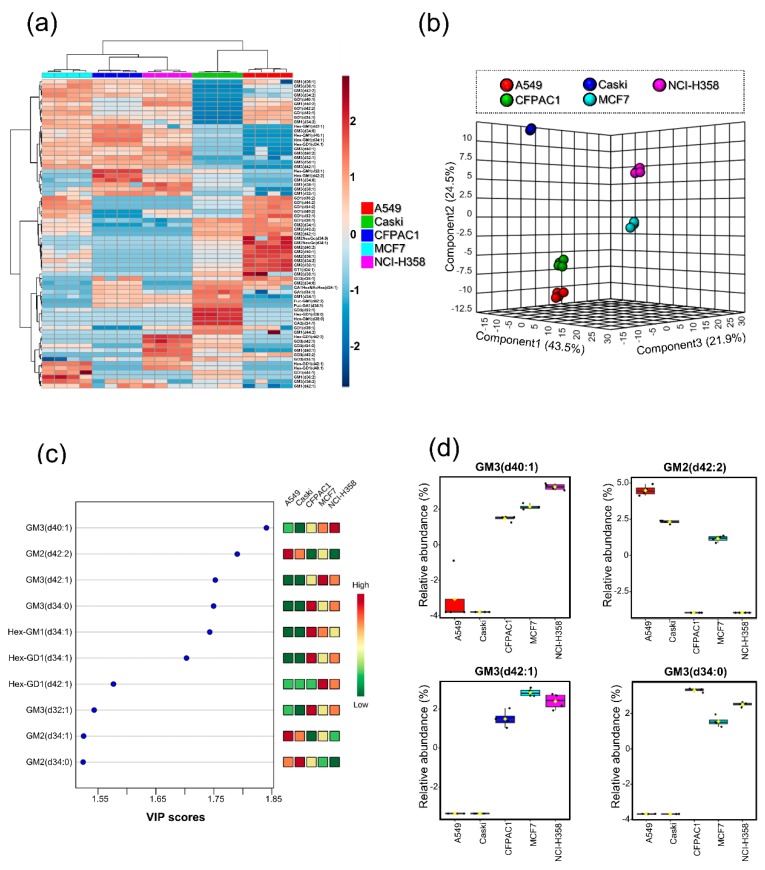
Statistical analysis to examine the relationship between cancer cell lines based on ganglioside distribution. (**a**) Heatmap illustrating the hierarchical clustering of cancer cell lines and 68 gangliosides profiles (ANOVA *p* < 0.05). Relative abundances of each gangliosides were standardized across samples. Scale bar indicates z-scores of standardized ganglioside values, with highly expressed gangliosides depicted in red and low-expressed gangliosides depicted in blue. Rows correspond to clusters obtained from total gangliosides. Cell line names have been color-coded as follows: A549 (red), Caski (green), CFPAC1 (blue), MCF7 (sky blue), and NCI-H358 (purple). (**b**) Partial least squares-discriminant analysis (PLS-DA) of cancer cell lines based on the relative abundances of total gangliosides with its 3-D indication. (**c**) Variable importance in projection (VIP) scores for ten gangliosides in order of significance (**d**) Boxplots of four significantly different gangliosides between cancer cell lines.

The relative abundance of individual ganglioside compositions was quantified in quadruplicates using the chromatographic peak area for overall quantitation of ganglioside from each cancer cell line. Then, we performed cluster analysis to investigate statistical relationship between the cell lines based on ganglioside profile (Figure 3a,b). All cancer cell lines were well-clustered in their replicates by the specifically expressed gangliosides. The haetmap in Figure 3a evidently showed that the qualitative and quantitative expression of gangliosides in each cell line was quite different. To examine important measures in PLS-DA (Figure 3b), variable importance in projection (VIP) scores were calculated (Figure 3c). Notably, mono-sialylated gangliosides bearing glycan heads, GM3 and GM2 were significantly different between cell lines. As shown in Figure 3d, gangliosides containing GM3 glycan head such as GM3 (d40:1), GM3 (d42:1), and GM3 (d34:0) were not detected in A549 and Caski, while they showed relatively high abundance in CFPAC1, MCF7, and NCI-H358. On the other hand, another mono-sialylated ganglioside, GM2(d42:2) was abundant in A549, Caski, and MCF7, while it was not detected in CFPAC1 and NCI-H358. Interestingly, lung cancer cell lines A549 and NCI-H358 were clearly distinguished according to ganglioside profile. In particular, A549 showed 4-fold elevating levels of di-sialylated gangliosides GD1 (d42:2) and GD1 (d34:1) than NCI-H358. Conclusively, we could distinguish five different cell lines using gangliosides expressed from each cell line. 

### 3.4. Ganglioside Differences between Cell Lines Comes from the Heterogeneity of Glycan Moiety 

**Figure 4:**
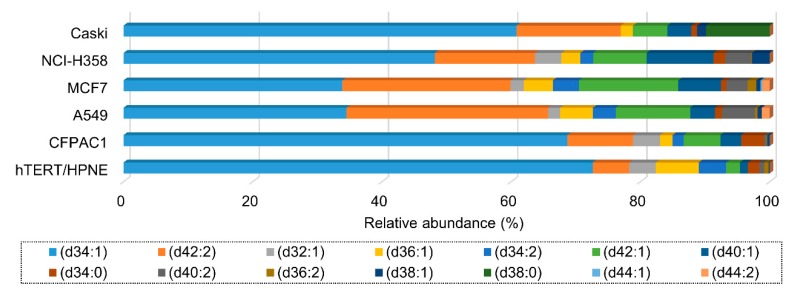
Relative abundances of gangliosides according to ceramide moieties in each cell line.

**Figure 5:**
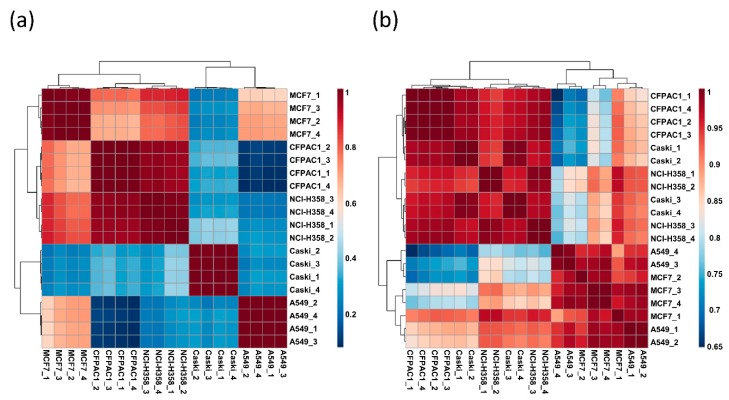
Heatmaps of pearson’s correlation coefficients (*r*) between cancer cell line samples based on (**a**) glycan portion and (**b**) ceramide portion.

Gangliosides consist of a hydrophobic ceramide portion, which anchors the ganglioside into the plasma membrane, and a hydrophilic glycan portion containing one or more sialic acids [35]. Gangliosides were reorganized separately into glycan and ceramide moiety in order to determine the differences between cell lines by which moiety. In specific, ganglioside abundance was recalculated according to 16 glycan heads found in each cell line (Table 1). Structurally simple glycan moieties such as GM1, GD1, and GM3 were generally abundant in all cancer cell line, but their abundance exhibited great diversity between cell lines. In particular, two subtypes of lung cancer, A549 and NCI-H358 were clearly differentiated by mono-sialylated gangliosides. GM2 was highly observed in A549 (more than 30% in relative abundance), while only a small amount was detected in NCI-H358 (less than 5% in relative abundance). On the other hand, GM3 was vice versa with GM2 in the A549 and NCI-H358. In addition, we examined the ceramide profiles by excluding the glycan moiety. The analysis of ceramide portion in each cell line is represented in Figure 4. The d34:1 and d42:2 were observed as major ceramide moieties in all cell lines, while they still showed difference in relative abundance. Furthermore, Pearson’s correlation analysis was performed using recalculated relative abundances of glycan and ceramide portion of total gangliosides in order to examine contribution of glycan and ceramide portion for distinctive ganglioside profile in each cancer cell line (Figure 5). Similarity between different cell lines was lower by glycan moiety (*r* = 0.084–1.000, Figure 5a), whereas higher by ceramide moiety (*r* = 0.661–1.000, Figure 5b), which indicates that glycan moiety was more heterogeneous depending on cell types than ceramide moiety. It is clearly demonstrated that glycan moiety in ganglioside is more closely associated with cell-type specific function in different cancers than ceramide moiety.

### 3.5. Ganglioside Differences between Cancerous and Noncancerous Cell Line

We additionally investigated ganglioside profiles in noncancerous pancreatic cell line (hTERT/HPNE) using an identical method in order to compare with cancerous pancreatic cell line (CFPAC1). Regardless of identical origin of the cell, pancreas, ganglioside distribution between hTERT/HPNE and CFPAC1 were quite different (Figure S4a,b). Three most abundant gangliosides accounting for more than 50% total relative abundance were GM1(d34:1), GD1(d34:1), and GD3(d34:1) in hTERT/HPNE, while GM3(d34:1), GM1(d34:1), and Hex-GM1(d34:1) in CFPAC1, indicating that differences in major ganglioside profiles between the cell lines may come from glycan moiety rather than ceramide moiety. The low correlation between CFPAC1 and hTERT/HPNE (*R*^2^ = 0.214) showed that the membrane ganglioside profiles of these two cell lines were apparently different (Figure S4c). In particular, abundance of GM3(d34:1) was approximately 5-fold higher in CFPAC1 (33%) than hTERT/HPNE (6%). Interestingly, GM3 was previously reported as a tumor-associated ganglioside antigen since it was overexpressed in various tumors, such as melanoma, while it showed lower amount in normal cells [36,37,38]. In addition, shorter chain gangliosides were reported to be mainly expressed in cancer cells compared to normal cells of the same origin in a previous study [39]. Interestingly, sialic acid in glyco-molecule is known to be closely related to cancer properties, including invasiveness and metastatic potential [38]. In addition, a sialidase NEU3 associated with plasma membrane is often upregulated in carcinogenesis, metastasis, and invasiveness, while it suppressed the apoptosis of cancer cell [40]. Thus, quantitative profiling data of gangliosides was reorganized according to number of sialic acid in order to in-depth examination of sialic acid in ganglioside. We observed that cancerous and noncancerous pancreatic cell lines could be differentiated according to sialic acid as shown in Figure S4d. Gangliosides from CFPAC1 cell line showed higher abundance of mono-sialylated glycan moiety compared to hTERT/HPNE. On the other hand, higher abundance of gangliosides containing di-, and tri-sialylated glycan moieties was the characteristic feature of hTERT/HPNE.

## 4. Discussion

Although refined classification and characterization of cancer cell types are essential to enhanced prediction of treatment responsiveness and development of targeted therapies [41], many cancer cell lines have still been poorly characterized. For example, targeted therapies for a type of triple negative breast cancers (TNBC; 15–20% of breast cancers) have not been developed due to the limitation of conventional subtyping of breast cancer in the clinic based on the expression of three main types of receptors, such as estrogen receptor (ER), progesterone receptor (PR) and the human epidermal growth factor receptor 2 (HER2, also known as ErbB2) [42]. Recently, a proteogenomic approach considering personal genomic variation as well as protein expression was performed in 105 breast cancer patients by Clinical Proteomic Tumor Analysis Consortium, but the subtyping of TNBC could not be achieved [43]. It emphasizes again the necessity for new approach to discover molecular target to address unmet needs in clinical research. 

Gangliosides readily change with physiological and pathophysiological processes such as cell growth, viral transformation, oncogenesis, or tumor progression [44,45]. Particularly, several tumor-associated gangliosides found in cancer cells, now have been suggested for cancer immunotherapy [36]. Furthermore, gangliosides have been considered to possess a potential to provide a solution for many unsolved parts for molecular characterization of cancer cell which cannot be explained by the glycoprotein research. For instance, although α2,6-sialyltransferase 1 (ST6Gal1) gene is inhibited in glioma cell lines, change in expression of α2,6-sialylated glycoprotein was not observed. However, specific glycan heads in gangliosides such as GM2a, GM3, GM1b, Gb3, and Gb4 were significantly altered in malignant glioma cell lines [46]. Therefore, the considerable potential as an anti-cancer therapeutic of gangliosides has generated markedly increasing interest in wide range of biomedical disciplines for decades. However, in contrast to glycoprotein, ganglioside analysis is still in its infancy. In addition, previous studies have been difficult to compare and contrast their outcomes between various cancer types, resulting in lacking of designating cancer type specific- ganglioside target. 

Thus, comprehensive structural profile of gangliosides using effective analytical platform should be the first step bridging the gap between descriptive analysis and mechanistic inquiry. However, many studies on gangliosides using conventional approach such as TLC could not provide comprehensive molecular profiling to date by being applicable to specific glycan epitopes. In this study, we utilized LC/MS/MS-based analytical platform to qualitatively and quantitatively identify unique cell surface, gangliosides at individual molecular level. We achieved the comprehensive profiling of intact gangliosides from membranes of various cell lines including different cancer cell subtypes and normal cell line and clearly demonstrated their differences for the first time using a highly reproducible analytical platform combining negative ion mode nano-LC/MS method with membrane enrichment technique. C18 based LC-MS enables separation of gangliosides with different degrees of unsaturation in their lipid portion [47]. Thus, cancer cell surface gangliosides were effectively separated on the basis of the chain length of the ceramides and the degree of saturation in LC/MS based analytical approach utilized in this study. Notably, we found that the diversity in gangliosides between cell lines was mainly caused by glycan moiety than ceramide moiety by structure specific LC-MS/MS approach. Interestingly, potential biomarkers for pancreatic cancer were found in a previous study on ganglioside from human serum using LC-MS [35]. Future study by larger cell line sample sets including diverse cancer subtypes and corresponding normal cell lines should be conducted to validate our result. However, the result on striking differences in surface gangliosides between cancer cells apparently suggested the feasibility of our platform as a new approach for future discovery of new target for cancer biomarker discovery as well as for discrimination of cell types. Thus, we believe that the ganglioside analysis using the described method could lead to a number of potential clinical applications.

## Figures and Tables

**Table 1:** Relative abundances of gangliosides according to glycan moieties in each cell line. Plus signs represent ganglioside expression levels from weak (+) to strong (++++) expression. (30%<: ++++; 30%>: +++; 15%>: ++; 5%>: +; ND: not detected).

	hTERT/HPNE	CFPAC1	A549	MCF7	NCI-H358	Caski
**GM1**	++++	+++	+++	+++	+++	++++
**GD1**	+++	++	++++	++++	++	+
**GD3**	++	+	+	+	+	+
**GM3**	++	++++	+	++++	++++	+
**GM2**	++	+	++++	+	+	++
**GA1**	+	+	+	+	+	+++
**GT1**	+	ND	+	+	ND	ND
**GD2**	+	ND	ND	ND	ND	ND
**Hex-GD1**	+	+	ND	+	+	+
**GT3**	+	ND	ND	ND	ND	ND
**GA1+Hexnac+Hex**	+	+	+	ND	ND	+
**Hex-GM1**	+	++	ND	+	+	++
**Fuc-GM1**	ND	+	ND	ND	+	+
**GM2(NeuGc)**	ND	ND	+	ND	ND	ND
**Fuc-GA1**	ND	+	ND	ND	+	++
**GA2**	ND	ND	ND	ND	ND	+

## Supplementary Materials

The following are available online at https://www.mdpi.com/2073-4409/8/11/1323/s1, Figure S1: LC-MS based workflow for investigation of gangliosides extracted from cancer cell surfaces, Figure S2: Comprehensive profiling of cancer cell surface gangliosides. (a) Representative extracted compound chromatograms (ECCs) of cell surface gangliosides detected in MCF7 cell line. (b) Magnified view of Figure S2a showing the wide dynamic range of our analytical platform. Representative tandem MS spectra of ganglioside [GM1(d34:1)] identified in MCF7 from (c) negative ion detection mode tandem MS (spectra at m/z 1516.83 [z = 1]) and (d) positive ion detection mode tandem MS (spectra at m/z 759.93 [z = 2]), Figure S3: Graphs of retention times (mins) vs. the number of carbons in the ceramides for different glycan head groups in all cell lines, Figure S4: Differences in ganglioside distribution between cancerous (CFPAC1) and noncancerous (hTERT/HPNE) cell lines. Pie charts indicating the content distribution of gangliosides accounting for 90% (relative abundance) of total gangliosides in (a) hTERT/HPNE and (b) CFPAC1. (c) Graphs of relative abundances of total gangliosides from CFPAC1 vs. hTERT/HPNE. (d) Relative abundances of gangliosides from CFPAC1 and hTERT/HPNE according to the number of sialic acids in their glycan heads, Table S1: A complete list of the ganglioside compositions and relative abundances.
