# Supplementary material for: Comprehensive Profiling of Surface Gangliosides Extracted from Various Cell Lines by LC-MS/MS
Source: Cells. 2019 Oct 26;8(11):1323. doi: 10.3390/cells8111323 (PMC6912501; doi:10.3390/cells8111323)
Supplement: Supplementary file 1 [file cells-08-01323-s001.pdf]

Supplementary Figures

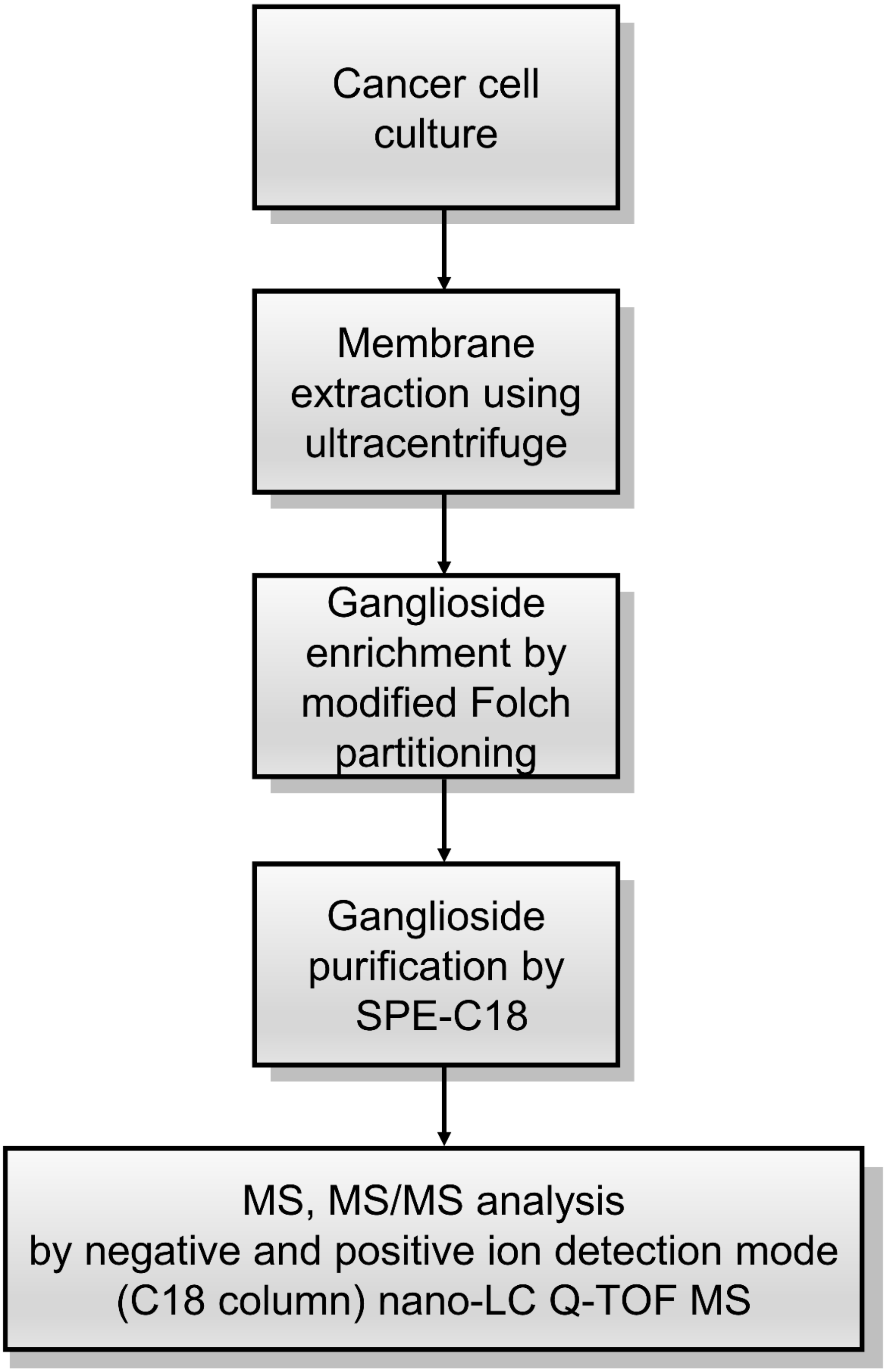

Figure S1: LC-MS based workflow for investigation of gangliosides extracted from cancer cell surfaces.

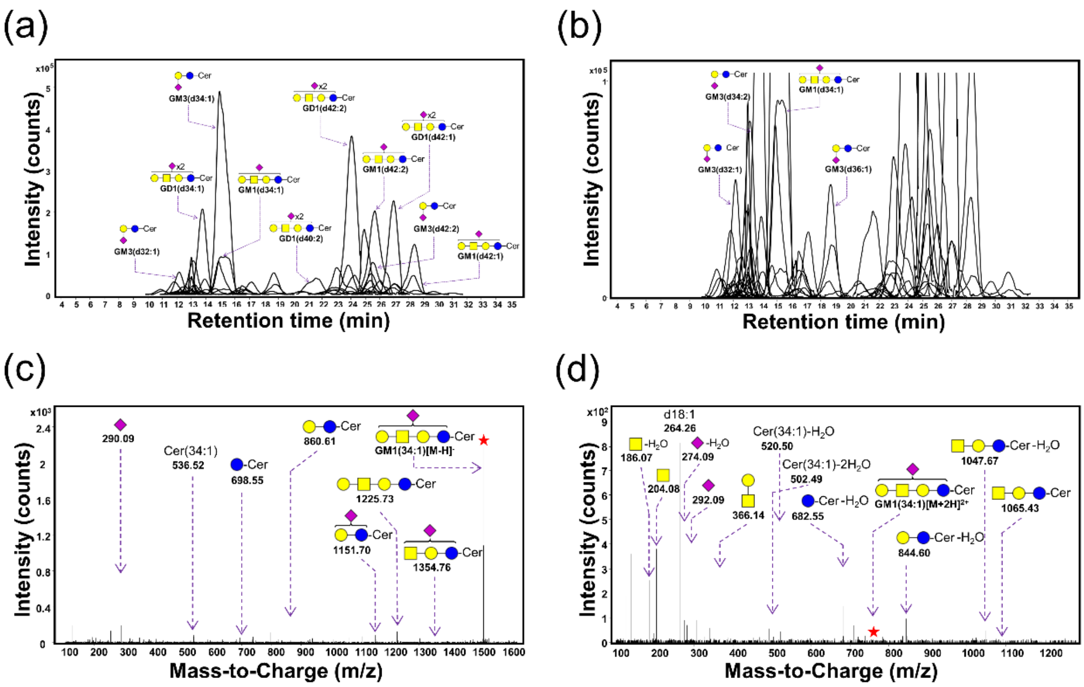

Figure S2: Comprehensive profiling of cancer cell surface gangliosides. (**a**) Representative extracted compound chromatograms (ECCs) of cell surface gangliosides detected in MCF7 cell line. (**b**) Magnified view of Figure S2a showing the wide dynamic range of our analytical platform. Representative tandem MS spectra of ganglioside [GM1(d34:1)] identified in MCF7 from (**c**) negative ion detection mode tandem MS (spectra at m/z 1516.83 [z=1]) and (**d**) positive ion detection mode tandem MS (spectra at m/z 759.93 [z=2]).

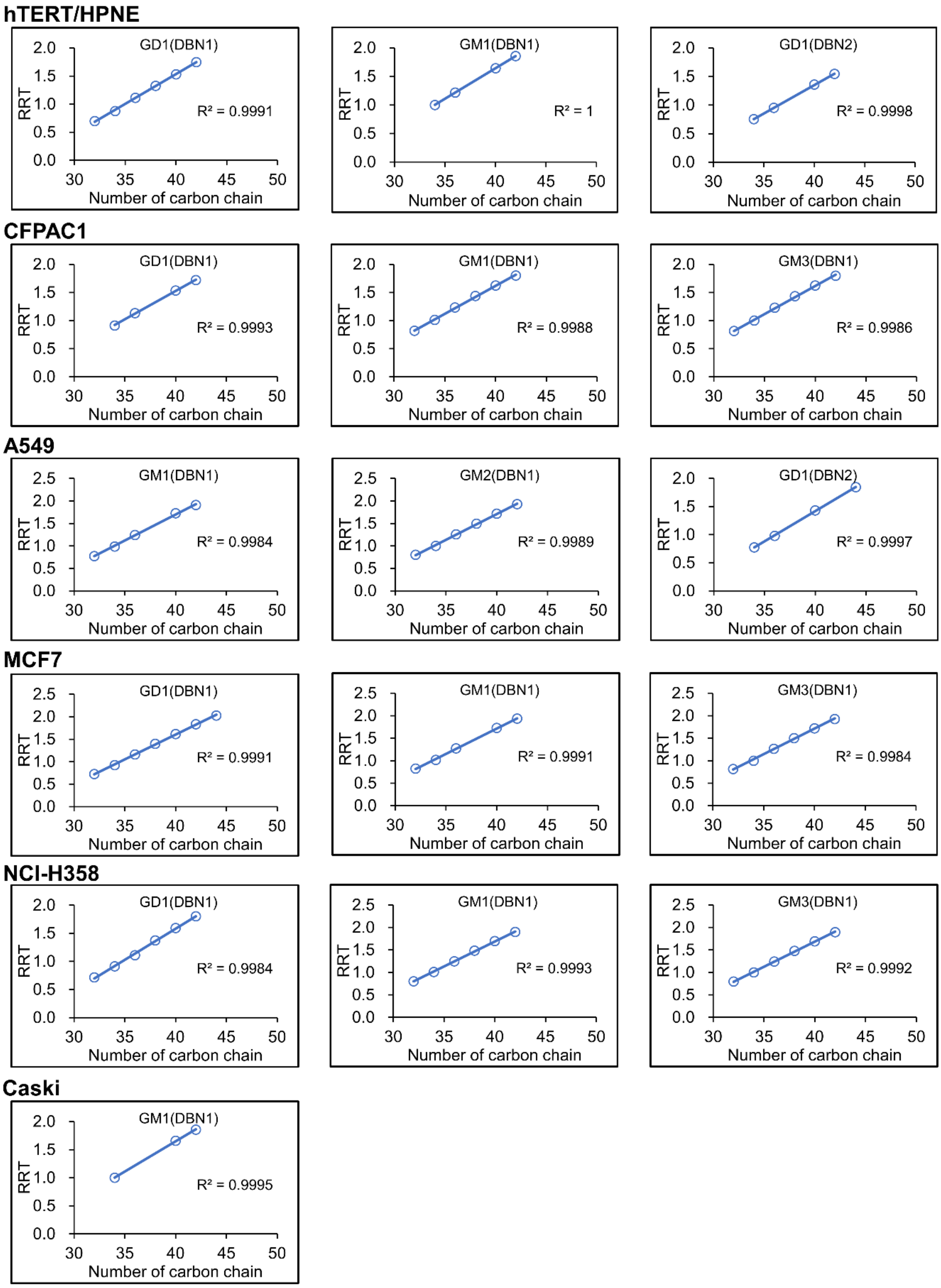

Figure S3: Graphs of retention times (mins) vs the number of carbons in the ceramides for different glycan head groups in all cell lines.

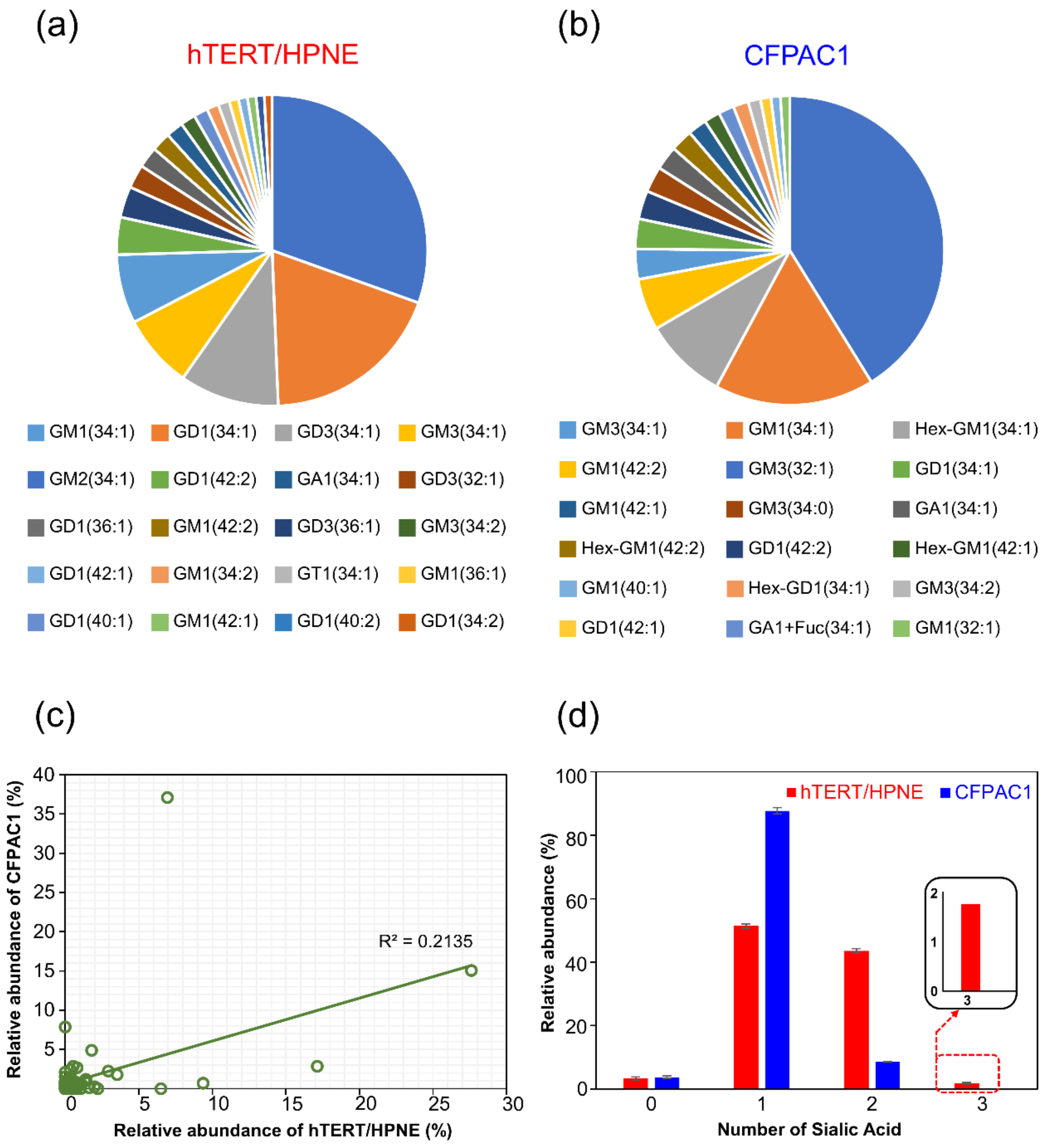

Figure S4: Differences in ganglioside distribution between cancerous (CFPAC1) and noncancerous (hTERT/HPNE) cell lines. Pie charts indicating the content distribution of gangliosides accounting for 90% (relative abundance) of total gangliosides in (**a**) hTERT/HPNE and (**b**) CFPAC1. (**c**) Graphs of relative abundances of total gangliosides from CFPAC1 vs hTERT/HPNE. (**d**) Relative abundances of gangliosides from CFPAC1 and hTERT/HPNE according to the number of sialic acids in their glycan heads.

Table S1: A complete list of the ganglioside compositions and relative abundances

Mass[M]	Ganglioside	hTERT/HPNE_1	hTERT/HPNE_2	CFPAC1_1	CFPAC1_2	CFPAC1_3	CFPAC1_4	A549_1	A549_2	A549_3	A549_4	MCF7_1	MCF7_2	MCF7_3	MCF7_4	NCI-H358_1	NCI-H358_2	NCI-H358_3	NCI-H358_4	Caski_1	Caski_2	Caski_3	Caski_4
1064.69709	**GA2(34:1)**	ND	ND	ND	ND	ND	ND	ND	ND	ND	ND	ND	ND	ND	ND	ND	ND	ND	ND	1.82952	1.72501	1.87544	1.82535
1124.68179	**GM3(32:1)**	0.50488	0.60492	3.34710	3.24770	2.28340	2.52204	0.16615	0.17590	ND	0.19587	1.32022	0.93159	1.40205	1.42592	2.35043	2.39932	3.54500	2.89447	ND	ND	ND	ND
1150.69744	**GM3(34:2)**	1.43182	1.38360	1.42884	1.43805	0.91762	0.89411	0.20943	0.20525	0.19927	0.26754	1.89819	1.35598	2.32047	2.58282	0.97891	1.02667	1.50844	1.41526	ND	ND	ND	ND
1152.71309	**GM3(34:1)**	7.54815	6.32574	38.04001	34.09552	35.86033	40.43861	1.48886	1.51072	1.47361	1.34703	22.20220	15.92539	17.20667	15.63532	27.15139	26.15199	33.25288	32.98993	2.44374	2.18680	1.99016	1.92580
1154.72874	**GM3(34:0)**	0.35949	0.35136	2.55604	2.51866	2.44312	2.20672	ND	ND	ND	ND	0.93758	0.69654	0.67430	0.57436	1.43841	1.40741	1.23298	1.51592	ND	ND	ND	ND
1178.72874	**GM3(36:2)**	ND	ND	0.13649	0.16167	ND	0.05813	ND	0.11112	ND	ND	0.49467	0.38972	0.85870	0.87389	0.20465	0.17113	ND	ND	ND	ND	ND	ND
1180.74439	**GM3(36:1)**	0.66138	0.66049	0.82648	0.91530	0.94033	0.81850	0.63843	0.57553	0.47440	0.39872	2.75912	2.86797	1.57981	1.34965	1.57301	1.34711	1.46694	1.39976	ND	ND	ND	ND
1208.77569	**GM3(38:1)**	ND	ND	0.15356	0.18157	0.21830	0.20250	0.07643	ND	ND	ND	0.28570	0.36905	0.21712	0.20105	0.89283	1.18810	1.04919	1.01124	ND	ND	ND	ND
1226.74989	**GA1(34:1)**	2.35632	3.49199	2.03077	2.16310	2.62124	2.18696	0.54283	ND	0.79457	0.61656	0.41803	0.22058	0.40597	0.37268	1.05697	0.76805	0.72412	1.01585	15.86729	15.99576	16.61570	15.74006
1234.79134	**GM3(40:2)**	ND	ND	0.10824	0.13074	0.14415	0.13618	ND	0.12731	ND	ND	0.49176	0.64386	0.66510	0.60999	0.92404	0.95619	1.18331	1.16836	ND	ND	ND	ND
1236.80699	**GM3(40:1)**	ND	ND	0.62209	0.76594	0.75655	0.76860	ND	0.14102	ND	ND	1.12991	1.32531	1.09919	1.04076	2.67378	2.86193	2.32435	2.18284	ND	ND	ND	ND

1262.82264	**GM3(42:2)**	ND	ND	0.69691	0.87781	1.06305	0.78168	0.12036	0.34591	0.41829	0.46010	2.24064	2.86341	2.85058	2.81290	1.67278	1.66637	1.86890	1.76608	ND	ND	ND	ND
1264.83829	**GM3(42:1)**	ND	ND	0.54240	0.79332	0.39549	0.50405	ND	ND	ND	ND	1.23777	1.64003	1.45407	1.22574	1.24312	1.30984	0.73466	0.87143	ND	ND	ND	ND
1327.76119	**GM2(32:1)**	0.60824	0.52357	ND	ND	ND	ND	1.09206	0.92019	1.18039	1.26120	0.13848	0.09707	0.19044	0.19217	ND	ND	ND	ND	ND	ND	ND	ND
1353.77684	**GM2(34:2)**	0.41904	0.61094	ND	ND	ND	ND	1.61235	1.37022	1.68950	2.02186	0.27330	0.20259	0.33430	0.39463	ND	ND	ND	ND	ND	ND	ND	ND
1355.79249	**GM2(34:1)**	7.13144	5.89603	ND	ND	ND	ND	14.33243	14.95705	12.79232	11.55686	2.16506	1.56886	2.30516	2.02155	0.21480	ND	0.29167	0.26751	9.73686	10.48492	6.75496	7.61431
1357.80814	**GM2(34:0)**	ND	ND	0.24710	0.22111	0.24188	0.25258	0.95793	0.51548	1.19395	0.78831	0.10749	ND	ND	0.06895	ND	ND	ND	ND	0.93288	1.11042	0.73859	0.84078
1371.78739	**GM2(NeuGc)(34:1)**	ND	ND	ND	ND	ND	ND	2.16778	1.22767	0.86362	1.87629	ND	ND	ND	ND	ND	ND	ND	ND	ND	ND	ND	ND
1372.80779	**GA1+Fuc(34:1)**	ND	ND	0.79457	0.82180	1.02622	0.89899	ND	ND	ND	ND	ND	ND	ND	ND	0.71060	0.48718	0.42682	0.64128	9.73476	9.89452	10.30965	9.76120
1373.80304	**GM2(NeuGc)(34:0)**	ND	ND	ND	ND	ND	ND	0.46669	ND	0.24304	0.32348	ND	ND	ND	ND	ND	ND	ND	ND	ND	ND	ND	ND
1383.82379	**GM2(36:1)**	0.50035	0.69180	ND	ND	ND	ND	2.61836	2.57796	1.82191	1.36662	0.35454	0.34911	0.27128	0.21014	ND	ND	ND	ND	ND	ND	ND	ND
1411.85509	**GM2(38:1)**	ND	ND	ND	ND	ND	ND	0.38017	0.53455	ND	ND	ND	ND	ND	ND	ND	ND	ND	ND	ND	ND	ND	ND
1415.77719	**GD3(32:1)**	2.45289	1.99221	ND	ND	ND	ND	ND	ND	ND	ND	0.05972	0.03768	ND	0.06222	ND	ND	ND	ND	ND	ND	ND	ND
1437.87074	**GM2(40:2)**	ND	ND	ND	ND	ND	ND	1.01824	1.56918	1.54893	1.48964	0.05434	0.12553	0.12008	ND	ND	ND	ND	ND	ND	ND	ND	ND
1439.88639	**GM2(40:1)**	ND	ND	ND	ND	ND	ND	1.25820	1.48673	1.65945	1.40990	0.22607	0.25455	0.26937	0.18533	ND	ND	ND	ND	ND	ND	ND	ND

1441.79284	**GD3(34:2)**	0.45660	0.49865	ND	ND	ND	ND	ND	ND	ND	ND	0.15340	0.10818	0.16763	0.19092	0.18602	0.19069	0.21238	0.18607	ND	ND	ND	ND
1443.80849	**GD3(34:1)**	10.20449	8.54968	0.84814	0.75043	0.66283	0.52844	1.33553	1.42256	0.73767	1.04717	0.67515	0.47221	0.30740	0.33211	1.71538	1.62310	1.70370	1.85713	1.63785	2.20551	1.51754	1.64927
1445.82414	**GD3(34:0)**	0.18422	0.13835	ND	ND	ND	ND	ND	ND	ND	ND	ND	ND	ND	ND	ND	ND	ND	ND	ND	ND	ND	ND
1465.90204	**GM2(42:2)**	ND	ND	ND	ND	ND	ND	5.86535	5.14728	6.94942	9.01007	0.53915	0.63001	0.71180	0.75385	ND	ND	ND	ND	1.54431	1.30746	1.54613	1.45653
1467.91769	**GM2(42:1)**	ND	ND	ND	ND	ND	ND	1.53300	1.44984	2.56048	0.85730	0.23928	0.37970	0.29517	0.34412	ND	ND	ND	ND	0.51251	0.46366	0.51004	0.61575
1469.82414	**GD3(36:2)**	0.33925	0.33537	ND	ND	ND	ND	ND	ND	ND	ND	ND	ND	ND	ND	ND	ND	ND	ND	ND	ND	ND	ND
1471.83979	**GD3(36:1)**	1.82071	1.44176	ND	0.20340	0.17432	0.12059	0.38102	0.36593	0.23600	0.22454	ND	ND	ND	ND	ND	ND	ND	ND	ND	ND	ND	ND
1489.81399	**GM1(32:1)**	ND	ND	0.92380	0.99352	0.67717	0.94292	0.13181	ND	0.24390	ND	0.43460	0.20350	0.22137	0.40360	1.28615	0.98521	1.39947	0.77789	ND	ND	ND	ND
1515.82964	**GM1(34:2)**	1.50613	0.73423	0.59116	0.64943	0.49261	0.39252	0.75244	0.42206	0.31354	0.81379	0.66885	0.47972	0.95312	0.82102	0.44302	0.46121	0.64728	0.70824	ND	ND	ND	ND
1517.84529	**GM1(34:1)**	25.67669	29.55495	15.31771	13.52237	15.72855	15.65986	5.77938	3.60936	2.55594	2.47028	6.36295	4.48477	5.68855	5.44191	10.01842	9.89856	8.39569	10.43155	19.99296	20.66560	15.76868	16.27454
1519.86094	**GM1(34:0)**	0.66709	0.52474	0.97974	0.79339	0.73565	0.93321	ND	ND	ND	ND	0.16396	0.10081	ND	0.10431	0.37924	0.41133	0.37974	0.56299	ND	ND	ND	ND
1543.86094	**GM1(36:2)**	ND	ND	ND	ND	ND	ND	ND	ND	ND	ND	0.31819	0.17036	0.40251	0.58867	ND	ND	ND	ND	ND	ND	ND	ND
1545.87659	**GM1(36:1)**	0.89421	0.87525	0.67136	0.75292	0.75257	0.60848	1.21497	0.84657	0.50968	ND	1.31797	1.36632	0.85864	0.74808	1.10945	1.08376	0.85958	0.71733	ND	ND	ND	ND
1553.91804	**GD3(42:2)**	0.26678	0.37830	ND	ND	ND	ND	0.26856	0.03816	0.29402	0.32287	ND	ND	ND	ND	1.09427	1.17101	1.14095	1.03458	ND	ND	ND	ND

1555.93369	**GD3(42:1)**	ND	ND	ND	ND	ND	ND	ND	ND	ND	ND	ND	ND	ND	ND	0.70457	0.84990	0.47445	0.49851	ND	ND	ND	ND
1573.90789	**GM1(38:1)**	ND	ND	0.20012	0.22501	0.24019	ND	ND	ND	ND	ND	ND	ND	ND	ND	1.08154	1.26434	0.71946	1.01105	ND	ND	ND	ND
1591.88209	**GA1+Hexnac+Hex(34:1)**	0.31507	0.41729	0.53756	0.61757	0.67715	0.52426	0.60213	0.63100	0.66176	ND	ND	ND	ND	ND	ND	ND	ND	ND	1.97910	2.76022	2.37879	2.26818
1599.92354	**GM1(40:2)**	ND	ND	0.25110	0.32696	0.35599	0.28021	0.39286	0.75453	0.67487	1.24705	0.58914	0.69979	0.77768	1.04065	2.04988	1.82829	1.77971	1.72699	ND	ND	ND	ND
1601.93919	**GM1(40:1)**	0.37688	0.37191	1.28196	1.56362	1.61303	1.43396	0.91551	1.09038	1.16654	1.15096	1.73783	2.04408	2.07932	1.71126	5.02056	5.59851	3.62101	3.64864	3.40542	2.54056	4.01826	4.73526
1627.95484	**GM1(42:2)**	1.29101	2.30516	3.67409	4.62831	5.78074	5.44040	7.03858	7.30619	10.62022	11.70535	5.92856	8.00572	8.05802	7.71068	5.59905	5.65023	5.39258	4.97267	9.67784	8.80557	13.76215	12.42397
1629.97049	**GM1(42:1)**	0.35042	1.26517	2.50458	3.97789	1.92749	2.29096	1.39873	2.08883	1.38082	1.43583	3.42446	4.69309	3.95205	3.90629	4.55899	4.01633	3.13603	2.75947	4.33387	3.77198	5.43123	5.77857
1651.86679	**Hex-GM1(32:1)**	0.15767	ND	0.39542	0.45681	0.48997	0.38980	ND	ND	ND	ND	ND	ND	ND	ND	ND	ND	ND	ND	ND	ND	ND	ND
1655.98614	**GM1(44:2)**	ND	ND	ND	ND	ND	ND	ND	ND	0.22195	ND	ND	ND	ND	ND	ND	ND	ND	ND	ND	ND	ND	ND
1674.91919	**GD2(36:1)**	0.59656	0.44865	ND	ND	ND	ND	ND	ND	ND	ND	ND	ND	ND	ND	ND	ND	ND	ND	ND	ND	ND	ND
1679.89809	**Hex-GM1(34:1)**	ND	ND	7.85048	8.26779	8.09509	7.23206	ND	ND	ND	ND	1.34379	0.91174	0.94263	1.41478	1.02212	1.01560	1.15557	1.13155	ND	ND	ND	ND
1734.90389	**GT3(34:1)**	0.35780	0.30896	ND	ND	ND	ND	ND	ND	ND	ND	ND	ND	ND	ND	ND	ND	ND	ND	ND	ND	ND	ND
1737.97634	**Hex-GM1(38:0)**	ND	ND	ND	ND	ND	ND	ND	ND	ND	ND	ND	ND	ND	ND	ND	ND	ND	ND	5.31845	5.30826	5.67998	5.83028
1763.99199	**Hex-GM1(40:1)**	ND	ND	0.53537	0.61193	0.62447	0.51023	ND	ND	ND	ND	0.30205	0.33763	0.31343	0.27370	0.41982	0.55012	0.33196	0.30530	ND	ND	ND	ND

1774.01274	**Fuc-GM1(42:2)**	ND	ND	0.42157	0.46010	0.63719	0.63033	ND	ND	ND	ND	ND	ND	ND	ND	1.05859	1.12012	0.89323	0.84938	3.11249	2.76799	3.99715	4.02466
1780.90939	**GD1(32:1)**	0.62604	0.67172	ND	ND	ND	ND	0.30918	0.38979	0.70580	0.73432	0.28127	0.18531	0.35813	0.38085	0.16108	0.18957	0.22096	0.20697	ND	ND	ND	ND
1790.00764	**Hex-GM1(42:2)**	ND	ND	1.85686	2.29750	2.66945	1.53717	ND	ND	ND	ND	ND	ND	ND	ND	0.68068	0.61769	0.62285	0.68125	ND	ND	ND	ND
1792.02329	**Hex-GM1(42:1)**	ND	ND	1.38719	1.90017	1.29496	1.39456	ND	ND	ND	ND	0.84459	0.86524	0.90368	0.96527	0.39462	0.46811	ND	0.30887	ND	ND	ND	ND
1806.92504	**GD1(34:2)**	0.57829	0.89650	ND	ND	ND	ND	0.88328	0.81008	1.33853	1.23075	0.72025	0.50651	0.98102	1.02731	ND	ND	ND	ND	ND	ND	ND	ND
1808.94069	**GD1(34:1)**	18.13750	16.13164	3.36541	3.27467	2.65445	2.04538	12.72504	13.90321	10.42093	10.98819	6.24686	4.26166	5.79930	5.61581	2.92429	2.84881	3.35230	3.41032	ND	ND	ND	ND
1810.95634	**GD1(34:0)**	0.71966	0.54324	ND	ND	ND	ND	ND	ND	ND	ND	ND	ND	ND	ND	ND	ND	ND	ND	ND	ND	ND	ND
1834.95634	**GD1(36:2)**	0.36810	0.35350	ND	ND	ND	ND	0.31792	0.28306	0.46272	0.40344	0.26800	0.19959	0.46192	0.47399	ND	ND	ND	ND	ND	ND	ND	ND
1836.97199	**GD1(36:1)**	2.20808	1.76194	0.27034	0.27971	0.22740	0.13909	1.63361	1.79901	1.21084	1.36192	1.17139	0.76140	0.94831	0.98769	0.53676	0.46598	0.51837	0.55285	1.86761	1.57701	2.06903	2.00560
1865.00329	**GD1(38:1)**	0.31048	ND	ND	ND	ND	ND	0.57674	0.54525	0.48715	ND	0.29918	0.39794	0.32552	0.30185	0.72884	0.71869	0.70907	0.66887	1.14680	1.30266	1.34263	1.76714
1891.01894	**GD1(40:2)**	0.71802	0.83019	ND	ND	ND	ND	2.67576	3.46617	3.36658	2.03287	1.32545	1.92827	1.99062	1.98687	0.99899	1.09167	1.19447	1.10162	ND	ND	ND	ND
1893.03459	**GD1(40:1)**	0.78984	0.90866	0.41352	0.44305	0.48181	0.43477	1.48705	1.35271	1.09188	1.07252	2.69740	3.29575	2.27826	2.25789	2.71041	2.86853	1.95423	1.82325	ND	ND	ND	ND
1919.05024	**GD1(42:2)**	3.39492	3.65050	1.56058	1.81774	1.97251	1.76077	14.28485	14.17461	14.07560	16.28823	12.45554	16.42907	14.89510	17.08628	4.35033	4.26112	4.23240	4.05090	ND	ND	ND	ND
1921.06589	**GD1(42:1)**	0.80110	1.86335	0.91020	1.17171	0.87288	1.00970	6.97675	8.45880	9.48048	8.48038	7.03448	10.00085	7.02051	7.16729	1.80790	3.03289	1.78568	1.44314	ND	ND	ND	ND

1947.08154	**GD1(44:2)**	ND	ND	ND	ND	ND	ND	1.00766	0.94416	1.41175	1.31559	0.91209	1.23978	1.19954	1.16270	ND	ND	ND	ND	ND	ND	ND	ND
1949.09719	**GD1(44:1)**	ND	ND	ND	ND	ND	ND	ND	ND	ND	ND	0.26111	0.46719	0.26146	0.29464	ND	ND	ND	ND	ND	ND	ND	ND
1970.99349	**Hex-GD1(34:1)**	0.42596	0.32293	1.72114	1.68172	1.24984	1.09068	ND	ND	ND	ND	1.08599	0.70234	1.11597	1.14411	0.92258	0.90131	1.12533	1.07217	ND	ND	ND	ND
2029.07174	**Hex-GD1(38:0)**	ND	ND	ND	ND	ND	ND	ND	ND	ND	ND	ND	ND	ND	ND	ND	ND	ND	ND	4.92574	5.12606	3.69388	3.46276
2055.08739	**Hex-GD1(40:1)**	ND	ND	ND	ND	ND	ND	ND	ND	ND	ND	0.36473	0.42400	0.32835	0.33097	0.74679	0.76770	0.53782	0.52840	ND	ND	ND	ND
2081.10304	**Hex-GD1(42:2)**	ND	ND	ND	ND	ND	ND	ND	ND	ND	ND	ND	ND	ND	ND	1.42890	1.48749	1.35505	1.33871	ND	ND	ND	ND
2083.11869	**Hex-GD1(42:1)**	ND	ND	ND	ND	ND	ND	ND	ND	ND	ND	0.85391	1.27821	0.95994	0.96162	0.77503	0.82082	0.53940	0.49342	ND	ND	ND	ND
2100.03609	**GT1(34:1)**	1.13166	0.98097	ND	ND	ND	ND	0.36557	0.34866	0.46766	0.42660	0.18362	0.10444	0.21840	0.22885	ND	ND	ND	ND	ND	ND	ND	ND
2128.06739	**GT1(36:1)**	0.35478	0.40381	ND	ND	ND	ND	ND	ND	ND	ND	ND	ND	ND	ND	ND	ND	ND	ND	ND	ND	ND	ND

.
